# The prediction of intraoperative cervical cord function changes by different motor evoked potentials phenotypes in cervical myelopathy patients

**DOI:** 10.1186/s12883-020-01799-w

**Published:** 2020-05-30

**Authors:** Shujie Wang, Zhifu Ren, Jia Liu, Jianguo Zhang, Ye Tian

**Affiliations:** 1 Department of Orthopedics, Peking Union Medical College Hospital, Chinese Academy of Medical Sciences & Peking Union Medical College, 1 Shuai Fu Yuan, Beijing, 100730 PR China; 2 Department of Spine Surgery, Municipal Traditional Chinese Hospital, Weifang, Shandong 261041 PR China; 3 China-America Institute of Neuroscience, Xuanwu Hospital, Capital Medical University, Beijing, 100053 China

**Keywords:** Cervical compressive myelopathy (CCM), Motor evoked potential (MEP), Intraoperative neuromonitoring, MEP baselines phenotypes, Intraoperative cervical cord function changes

## Abstract

**Background:**

Surgery is usually the treatment of choice for patients with cervical compressive myelopathy (CCM). Motor evoked potential (MEP) has proved to be helpful tool in evaluating intraoperative cervical spinal cord function change of those patients. This study aims to describe and evaluate different MEP baseline phenotypes for predicting MEP changes during CCM surgery.

**Methods:**

A total of 105 consecutive CCM patients underwent posterior cervical spine decompression were prospectively collected between December 2012 and November 2016. All intraoperative MEP baselines recorded before spinal cord decompression were classified into 5 types (I to V) that were carefully designed according to the different MEP parameters. The postoperative neurologic status of each patient was assessed immediately after surgery.

**Results:**

The mean intraoperative MEP changes range were 10.2% ± 5.8, 14.7% ± 9.2, 54.8% ± 31.9, 74.1% ± 24.3, and 110% ± 40 in Type I, II, III, IV, and V, respectively. There was a significant correlation of the intraoperative MEP change rate with different MEP baseline phenotypes (*r* = 0.84, *P* < 0.01). Postoperative transient new spinal deficits were found 0/31 case in Type I, 0/21 in Type II, 1/14 in Type III, 2/24 in Type IV, and 4/15 in Type V. No permanent neurological injury was found in our cases series.

**Conclusions:**

The MEP baselines categories for predicting intraoperative cervical cord function change is proposed through this work. The more serious the MEP baseline abnormality, the higher the probability of intraoperative MEP changes, which is beneficial to early warning for the cervical cord injury.

## Background

Cervical compressive myelopathy (CCM) is one of the most commonly acquired cause of spinal cord dysfunction [1], and surgery is usually the treatment of choice for those patients. It is important to assess cervical cord function in patients with CCM during surgical treatment. Over the past decades, the majority of studies are concerning the application of intraoperative transcranial motor evoked potential (MEP) to detect impending spinal cord damage, early warning the operating team to take action to avoid injury in cervical spine surgery [2–7].

Previously, imaging methods such as magnetic resonance imaging (MRI) are able to detect pathologic changes in patients with CCM, and are thought to be useful for the evaluation of prognosis [8–12]. Another reported predictor of postoperative prognosis is the preoperative cross-sectional area of the spinal cord at the site of maximum compression [13–15]. furthermore, somatosensory-evoked potentials (SEPs) classification system could be used as an objective tool, in addition to clinical scales, for the quantitative assessment of spinal cord function in CCM [16]. These are preoperative prediction methods for postoperative spinal cord function change.

Currently, we have found that the different intraoperative MEP baselines before cord decompression probably can predict the impending neuromonitoring changes after spinal canal decompression. In order to further test this hypothesis, our objective was to put forward different intraoperative MEP phenotypes and verify its feasibility for predicting the neuromonitoring changes after spinal decompression in CCM patients.

## Methods

### Patients

**Fig. 1:**
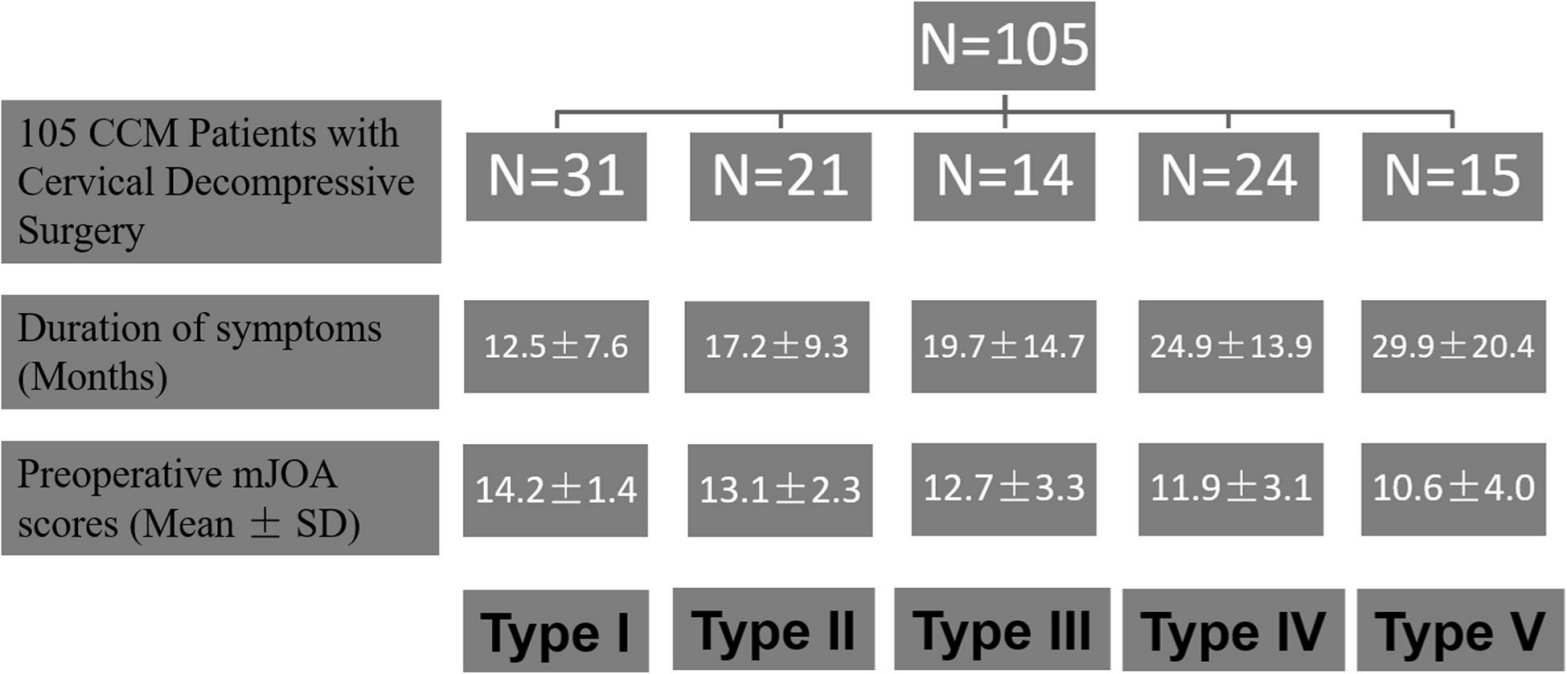
Patients’ duration of symptoms and preop mJOA scores associated with MEP classification

We prospectively collected 105 consecutive patients from December 2012 to December 2016. The major clinical characteristics and diagnosis of the population were showed in Table 1. Patients were eligible for this study when they met all the following inclusion criteria: (1) presenting with symptomatic CCM with at least 1 clinical sign of myelopathy; (2) evidence of objective CCM on a magnetic resonance image (MRI); (3) absence symptomatic lumbar stenosis or thoracic myelopathy; (4) no previous surgical treatment for CCM; (5) undergoing the same anesthesia regime. Patients’ duration of symptoms and preop modified Japanese Orthopedic Association (mJOA) scores associated with MEP phenotypes were showed in Fig. 1.

**Table 1:** The general data and the clinical diagnoses of all patients

General data and diagnosis	Mean ± SD (Range)/N (%)
**General data**	
Age	58.2 ± 11.1 (26–75 y)
Male/Female	71/34
Height	168.9 ± 8.4 (140–180 cm)
Weight	71.4 ± 14.1 (42–98 kg)
BMI	24.9 ± 4.1 (15–34)
Operation time	164.8 ± 39.3 (110–300 min)
Bleeding volume	232.2 ± 179.5 (150–900 ml)
**Diagnosis (n, %)**	
Cervical spondylotic myelopathy	63 (60.0%)
OPLL	13 (12.4%)
Cervical disk herniation	6 (5.7%)
Congenital anomaly of cervical spine	4 (3.8%)
Atlantoaxial subluxation	12 (11.4%)
Others	7 (6.7%)
**Type of surgery (n, %)**	
ACDF	19 (18.1%)
PCDF	78 (74.3%)
Laminectomy	8 (7.6%)

OPLL Ossification of posterior longitudinal ligament, ACDF Anterior cervical decompression and fusion, PCDF Posterior cervical decompression and fusion

### Preoperative and postoperative neurological assessment

All patients underwent a detailed preoperative assessment of neurological function and the degree of cord compression on MRI with MEP baseline recording. The postoperative neurologic status of each patient was assessed immediately after surgery by comparing the patient’s documented preoperative motor and sensory function with his or her early postoperative motor and sensory function.

### Intraoperative electrophysiological assessment

Two kinds of monitoring instruments (Cadwell Industries Inc. Cascade Pro Systems, Kennewick, WA, USA; Axon Systems Inc., Hauppauge, NY) were used. Moreover, MEP testing was performed after showing the vertebral lamina using subcutaneous needle electrodes by stimulating of 400 V constant voltage and multiple trains of 6 pulses, with duration of 400 μs. The inter-stimulus interval was 2.5 ms for each stimulation trains. The two pairs of stimulation electrodes were inserted subcutaneously over motor cortex regions C3–C4. Recording electrodes were placed into the abductor hallucis muscles in both of the lower extremities and the first dorsal interosseous muscles in the upper extremities (control).

According to many previous studies [17] and our experience [18–21], in cervical compressive myelopathy (CCM) patients underwent neural decompressive surgery, the surgical induced MEP alerting often derived from the procedure of spinal decompression especially severe spinal oppression segment. Otherwise it is often physiological or false-positive and we should first rule out systemic and anesthetic factors first [22]. The MEPs could be read directly from our monitoring instrument in every surgical point and were collected by averaging three times.

### The 5 MEP types

**Fig. 2:**
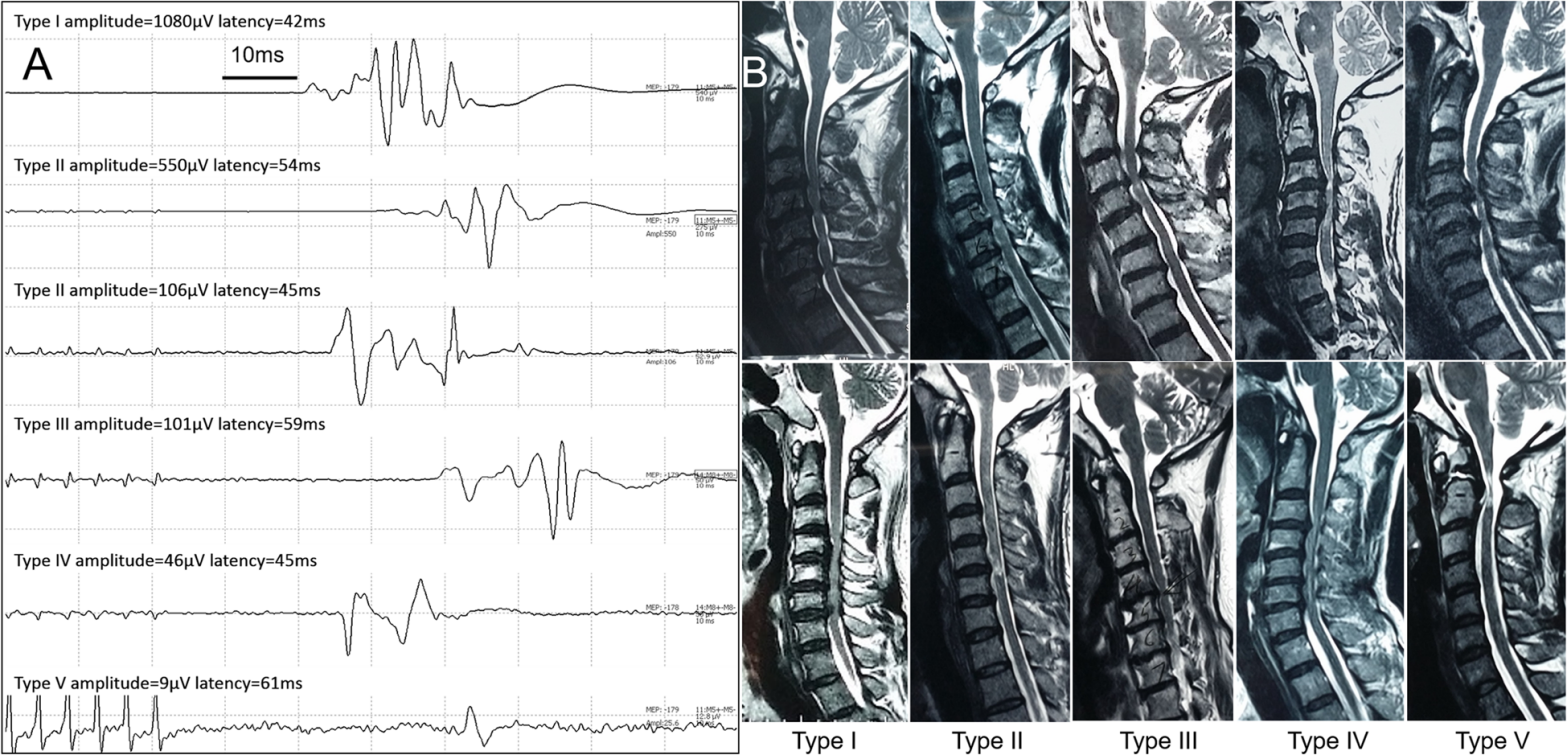
**a** The representative MEP waveform and parameters among the 5 baseline types. Type II has two subtypes. **b** The preoperative T2-weighted sagittal magnetic resonance image (MRI) from the patients with different MEP baseline types (Types I, II, III, IV and V)

In current study, a different MEP phenotype (Table 2) was firstly addressed for predicting the IONM changes for CCM patients e.g. in Fig. 2.

**Table 2:** The detail of different MEP baseline phenotypes

Types	Amplitude (μV)	Latency (ms)	Stability
**I**	> 300	< 50	Excellent
**II**	> 300	> 50	Good
	100–300	< 50	
**III**	100–300	> 50	Moderate
**IV**	20–100	–	Poor
**V**	0–20	–	Indeterminate/High Risk

The MEP types in this study aimed at unilateral baseline

Type I indicated safe MEP baseline with excellent stability;

Type II also indicated safe MEP baseline with good stability;

Type III was referred to sensitive MEP baseline with average stability;

Type IV was high-sensitive MEP with poor stability;

Type V was indeterminate/high risk MEP baseline.

The MEPs were recorded from the foot muscles (abductor hallucis) for the classification. The MEP types in this study aimed at unilateral baseline, amplitude and latency were considered as peak to peak and the initial of trains’ stimulation to the initial of MEP response respectively. And the baseline classification in Table 2 relies on the MEP stability that is defined based on the amplitude and latency.

### Anesthesia management

General anesthesia was induced with a bolus dose of propofol (3 mg/kg) and fentanyl (2.5 μg/kg) combined with a short-acting muscle relaxant (rocuronium) and inhalation agents (sevoflurane or nitrous oxide). Subsequently, maintenance of anesthesia was propofol (5–8 mg/kg/h) based on hemodynamic response; remifentanyl (0.05-2μg/kg/min); and a total dose of 5–6 μg/kg fentanyls (intermittent infusion) were used. No muscle relaxant or inhalation agent was used after anesthesia induction.

**Fig. 3:**
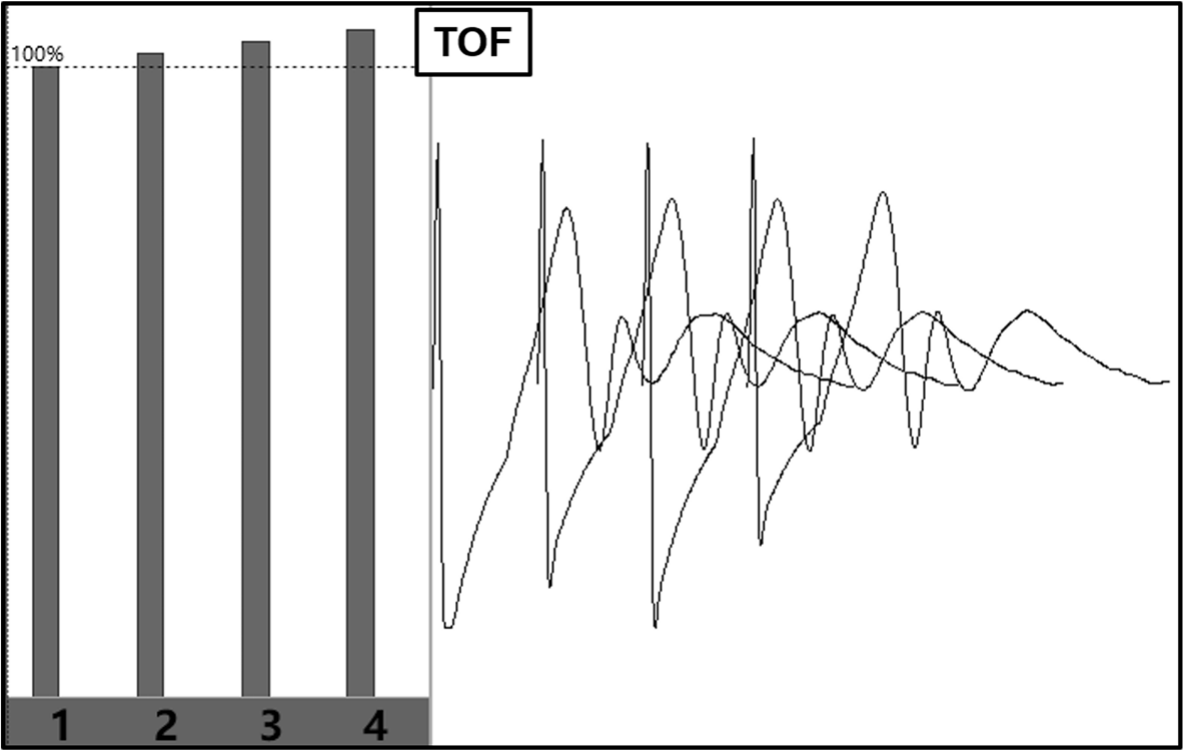
The TOF were monitored when recording MEP baseline

It should be noted that anesthetic is crucial role for MEP recording and classification, so all consecutive patients underwent the same anesthesia regime. MEP baseline must be recorded after performing the rocuronium of more than 50 min [23]. The patients with different TOF might affect the MEP responses, even using total intravenous anesthesia (TIVA), thus within 100 ± 5% TOF changes were confirmed when recording MEP baseline (Fig. 3). Moreover, the electroencephalographic density spectral array was also used to evaluate the depth of anesthesia, and we also strictly controlled the BIS value as 50–60 when recording the MEP signals.

### Statistical analysis

General data of patients were described as means and SD or n (%), and the statistical analyses were performed using Microsoft Excel 2007 (Microsoft, Redmond, WA, USA) and SPSS 19.0 (SPSS, Inc., Chicago, IL, USA) software. Statistical comparisons were made by χ^2^ test, and *P* < 0.05 was considered significant. Pearson’s correlation test was applied to evaluate the correlation of MEP phenotypes with the incidence of intraoperative monitoring changes.

## Results

**Fig. 4:**
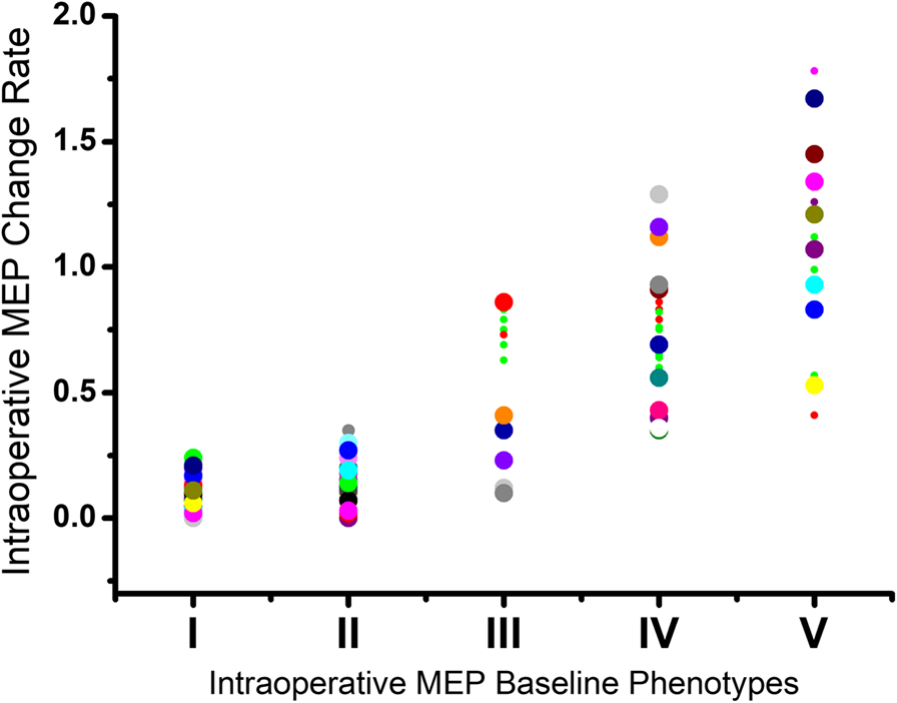
Absolute value distribution of intraoperative MEP change rate with different baseline phenotypes

Our results showed that 31 cases were classified as type I, 21 as type II, 14 as type III, 24 cases as type IV, and 15 cases as type V. The mean MEP changes range were 10.2% ± 5.8, 14.7% ± 9.2, 54.8% ± 31.9, 74.1% ± 24.3, and 110% ± 40 in Type I, II, III, IV, and V, respectively. With the MEP types proceeding, the neuromonitoring changes is increasing. And there was a significant correlation of the intraoperative MEP change rate with different MEP baseline classification (*r* = 0.84, *P* < 0.01) (Fig. 4).

No significant neuromonitoring change and postoperative new spinal deficit was found in Type I & II. Among 14 patients in Type III, 2 showed significant MEP losses, 5 showed intraoperative MEP improvements. Among 24 patients in Type IV, 4 showed significant MEP loss, 6 showed MEP improvements. Among 15 patients in Type V, 7 showed significant MEP loss, 6 showed MEP improvements (Table 3). Postoperative new spinal deficits were found 0/31 case in Type I, 0/21 in Type II, 1/14 in Type III, 2/24 in Type IV, and 4/15 in Type V. Furthermore, Multivariate analysis indicated that these three variables (Duration of symptoms, Preoperative SC cross-sectional area, MEP baseline categories) were also the main significant contributors for the impending MEP warning**.** (Table 4).

**Table 3:** The summary of monitoring changes and new neurologic deficit for each MEP baseline type from 105 CCM patients

	Type I	Type II	Type III	Type IV	Type V
**Significant monitoring loss**	0	0	2	4	7
**Significant monitoring improvement**	0	1	5	6	6
**New neurologic deficits**	0	0	1	2	4
**Total cases (%)**	31 (29.5%)	21 (20.0%)	14 (13.3%)	24 (22.9%)	15 (14.3%)

**Table 4:** Results of stepwise multivariate regression analysis

***Variable = β***_***0***_	MEP warning	
	Model: (R^2=^0.301, *p*<0.01)	
	β-coefficient	*P* Value
*Duration of symptoms*	0.205	0.0081
*Preoperative mJOA score*	0.018	0.0358
*Preoperative SC cross-sectional area*	0.189	0.0095
*MEP baseline categories*	0.268	0.0026

Note: All independent variables were entered into the regression. Values denoted are β-coefficient values (95% confidence intervals)

*mJOA* Japanese Orthopedic Association, *MEP* Motor-Evoked Potentials, *SC* Spinal Cord

## Discussion

The present study had focused on describing different MEP phenotypes for predicting intraoperative neuromonitoring change in patient underwent cervical cord decompression surgery. The main finding of this study was that the MEP baselines were classified into 5 types to imply the possibility of monitoring changes in different level during CCM surgery. The type I and II are safe and stable MEP baseline that generally does not present significant intraoperative monitoring changes; type III and IV are sensitive MEP baseline that should attract our attention for possible monitoring changes; type V is high-risk MEP baseline that would imply a possibility of great neuromonitoring changes. The main clinical significance of this knowledge was that we provide a novel predictor to help surgeons further assess the possible changes of cervical cord function, identify high-risk patients and then institute rigorous prevention strategies to achieve safer and more secure treatment for CCM patients.

The rationale for MEP monitoring is to directly test the integrity of lateral corticospinal tract and cervical nerve roots and then assess the function of motor system during cervical spine surgery [24, 25]. And MEPs amplitude change is highly sensitivity (approaching 100% & specificity (more than 95%) [22, 26, 27]) to predict a new postoperative neurological injury. Thus, MEP baseline signal may indicate the impairment’s degree of cervical cord or motor system dysfunction in pure CCM patients. During the patients with the type I or II MEP baseline, the injuries degree of nervous tissue from blood supply or direct mechanical cord compression are probably in relative compensatory stage.

On the other hand, the accumulated experimental evidence suggests that reperfusion lesions can result in neuronal death, which reactive oxygen radicals have been implicated to play an important role after decompression of a chronic compressive lesion of the cervical cord [28–30]. And reperfusion can occur in any level and any spot where surgical decompression was performed for the chronic compressive lesion. The severity and area of the reperfusion lesions depend on the area where neurons are really damaged. Theoretically, the neurons from cervical cord cannot present the high-risk reperfusion lesions in those patients with type I and II MEPs baseline. Meanwhile our current data can also support this point. The 52 patients with type I and II MEPs baseline did not appear significant intraoperative monitoring loss or new spinal deficit. Therefore, the type I and II MEPs baseline is safe and will usually not show an impending significant monitoring loss and then to predict cervical cord injury during the surgery of CCM.

Next, the CCM patients with Type III and IV MEPs baseline often have severe and long-term preoperative cervical cord compression and then perform poor MEP baselines. According to our data and experience, the monitoring signals often change in the patients with these baseline types. Interestingly, the patients with Type III or IV baseline not only perform a monitoring loss but also lots of monitoring improvement cases (Table 3). In our opinion, the mainly reason for monitoring loss is probably related to neural reperfusion lesions injury; the monitoring improvement is probably because the excitability of neuron or corticospinal tract is improving following cervical cord decompression. And the increasing arterial supply would also improve spinal cord ischemia and then benefit the MEP augment. Moreover, many previous studies have also proved that in patients with preoperative compression of the spinal cord or cauda equina, resulting in low or absent monitoring at the start of surgery, an immediate increase in MEPs after surgical decompression may predict a recovery of neurologic outcomes [31–34]. Furthermore, during our finding, only when the MEP baseline was in the Type III or IV, the amplitude would change easily and frequently and we should be careful that ahead of the cord decompression. Thus, the MEP in Type III or IV is high-sensitive baseline that should really be aroused our attention.

The Type V often derives from the patients with preoperative severe cervical cord compression, which often present sporadic disappearance or sudden enlargement without high-risk surgical maneuvers. On the basis of previous study, [35–38] increasing degrees of spinal cord deficits were associated with depressed feasibility of intraoperative MEP/SEP monitoring, respectively. Moreover, according to our data and experience, the Type V MEP baseline is really very difficult to accurately predict a new neurologic deficit, but when it is constant or amplifying after cervical cord decompression that usually implies a favorable prognostic in CCM patients.

MEPs reflect spinal cord functional status, especially pathologic changes in the entire corticospinal motor system below the brain stem [39–45]. The type III, IV, and V MEPs baseline can often gradually reflect the severity of clinical symptoms. Some previous study also showed a correlation between the mJOA score and the electrophysiological parameters in CCM [7, 18, 19, 46]. Moreover, according to this study and previous report [47], the cervical cord morphology and electrophysiological parameters are also relevant in CCM. Therefore, we can draw the conclusion that there are significant correlations among cervical cord morphology, electrophysiological parameters and clinical features. As thus we can use the MEP classification to represent the quantitative neurologic function and provide the high-sensitive MEP types to help us to predict the monitoring change ahead in decompressive CCM surgery.

### Limitations

There are some limitations must be clarified in this study. First, the MEP baseline classification is derived from our hypothesis and experience of a single institution. Further studies enrolling larger number of cases and a multicenter prospective evaluation of the reliability and validity of this new classification are needed. Second, although we believe our analysis to be compelling, the new classification system is still in the stage of exploration and appropriate corrections are much needed in the future. Third, because of the number of patients in this study was relatively small, the postoperative new spinal deficit among the 5 types did not present statistical significance (Table 3). But the correlations between the MEP baseline and the postoperative spinal deficit are appearing in our follow-up study of large sample cases.

## Conclusions

In this paper, we exhibited different MEP phenotypes before surgical spinal decompression during CCM patients. Using this MEP classification, the more serious the MEP baseline abnormality, the higher the probability of intraoperative monitoring changes. That is probably help surgeons to predict the impending spinal cord function changes in CCM patients.

## Data Availability

All data generated or analyzed during this study are included in this published article.

## Abbreviations

CCM: Cervical Compressive Myelopathy
MEP: Motor Evoked Potential
mJOA: Modified Japanese Orthopedic Association
TIVA: Total Intravenous Anesthesia
